# Momentary Conscious Pairing Eliminates Unconscious-Stimulus Influences on Task Selection

**DOI:** 10.1371/journal.pone.0046320

**Published:** 2012-09-25

**Authors:** Fanzhi Anita Zhou, Greg Davis

**Affiliations:** Department of Experimental Psychology, University of Cambridge, Cambridge, United Kingdom; University Of Cambridge, United Kingdom

## Abstract

Task selection, previously thought to operate only under conscious, voluntary control, can be activated by unconsciously-perceived stimuli. In most cases, such activation is observed for unconscious stimuli that closely resemble other conscious, task-relevant stimuli and hence may simply reflect perceptual activation of consciously established stimulus-task associations. However, other studies have reported ‘direct’ unconscious-stimulus influences on task selection in the absence of any conscious, voluntary association between that stimulus and task (e.g., Zhou and Davis, 2012). In new experiments, described here, these latter influences on cued- and free-choice task selection appear robust and long-lived, yet, paradoxically, are suppressed to undetectable levels following momentary conscious prime-task pairing. Assessing, and rejecting, three intuitive explanations for such suppressive effects, we conclude that conscious prime-task pairing minimizes non-strategic influences of unconscious stimuli on task selection, insulating endogenous choice mechanisms from maladaptive external control.

## Introduction

Our choices are often accompanied by a clear subjective sense of conscious autonomy and an independence from immediate control by environmental stimuli. This perceived freedom is particularly salient in cases of unspeeded, uncued choices but is also evident in choices that are instructed by environmental stimuli (e.g., the choice whether or not to obey a speed-limit sign). In direct contradiction of this subjective autonomy a broad consensus in the neurosciences holds that choices are determined by unconscious processes, including those driven by unconsciously-perceived, external stimuli.

Current neuroscientific interest in choices that feel ‘free’ stems largely from Libet's initially unpopular, yet pioneering work using physiological markers to predict choices prior to a participant's own awareness of the ‘urge’ to choose a particular action [1]. The influence of this work was greatly enhanced following Haggard and Eimer's (1999) discovery that lateralized readiness potentials correlated with and predicted conscious choices and subsequent studies have extended this approach to attempt trial-by-trial predictions of free-choices on the basis of physiological markers of unconscious processing [2]. Such predictions exploit natural co-variation in physiological markers of unconscious processes and verbal reports of conscious choices to infer that the former cause the latter. However, the nature of this relationship is far from transparent and has been recently challenged [3]. Schurger and colleagues' experiments and accumulator model suggest that it is only an indirect relationship, mediated by other processes. Moreover, such correlative procedures are conceptually limited in that they cannot distinguish endogenous, unconscious initiation of ‘free willed’ choices [4] from external control of choices postulated by more radical, ‘illusionist’ perspectives [5]. This latter debate centres on the degree of control that unconsciously-perceived stimuli in our immediate environment can control choices, not as a function of rendering one choice more attractive than another [6], [7] but rather by directly influencing choice mechanisms. Accordingly, some recent work has adopted an alternative approach that promises to reveal more directly the origins of control over our choices.

Two widely cited studies of this latter more direct approach have employed sub-threshold trans-cranial magnetic stimulation (TMS) of motor, frontal and parietal cortices to prime participants' choices of which hand they responded with, sometimes reporting effects even when participants thought they were making ‘free’ choices [8], [9]. These effects are small and do not always generalise across tasks. TMS to frontal cortex induced a 5–6% bias in one paradigm [9], but in a related task was only found for extremely rapid (<200 ms), reflexive movements and only then when motor cortex itself was stimulated [8]. More robust influences, however, have been reported by a range of different studies in which unconsciously-perceived visual stimuli influence which of two responses a participant will choose and the speed of their responses are evident [10]–[12]. These findings suggest that there is essentially a porous boundary between environmental stimuli and brain mechanisms involved in choices, such that the former exert unfettered control over the latter.

One potential objection to all of the above studies is that they examine choices as to which of two keys to press [13], a simple choice that may be relatively more under unconscious control than more complex or longer-term choices. This objection is partially addressed by Lau and Passingham (2007) and Reuss, Kiesel, Kunde and Hommel (2011) who have demonstrated unconscious-stimulus influences over preparation to perform a task and over ‘free’ task choice. Lau and Passingham (2007) asked participants on each trial to perform one of two word-related tasks as indicated by one of two conscious instruction shapes (either a diamond or a square). Under these conditions, the instruction consciously triggers activation of a ‘task set’ – the mental preparation to perform one task and inhibit responses associated with another [14], [15]. The instruction in Lau and Passingham's task appeared only 150 ms prior to the target word, ensuring that necessary mental preparations to perform the task (i.e., to establish a particular dominant ‘task set’) would be incomplete when the word appeared. An unconscious prime stimulus (again a diamond or square) was also presented shortly prior to the instruction: on half the trials this unconscious stimulus was congruent with the instruction in that it signalled the same task, and on the other half it was incongruent with the instruction, signalling the other task. Responses to the word tasks were faster when prime and instruction were congruent (i.e., signalled the same task) than when they were incongruent (signalled different tasks) suggesting that the unconscious primes had activated task sets. That is, the external, unconscious stimulus had caused the participant to begin mentally preparing to perform a task in a manner that was not under their voluntary conscious control. Reuss and colleagues (2011) have subsequently extended Lau and Passingham's paradigm, demonstrating that unconscious stimuli can reliably bias participants' free and unhurried choice of which task they would perform on a given trial.

Though these studies extend previous findings of unconscious control of choices to task selection, they share with previous work a limitation that limits the degree to which they can speak to external control of choices. In all the previous work described above, participants consciously and voluntary established an association between each shape and its associated task (or, an associated response in studies examining simpler choices). Accordingly, conscious knowledge of the prime-task relationships might have been necessary to observe effects of unconscious primes. Furthermore, since the primes that might appear on a given trial were the same two shapes that the participant must actively try to detect and recognize as conscious task instructions, they would have had an active attentional set to search for those shapes [16]. Indeed, in those studies, the participant agrees to respond by performing task A whenever they see, for example, a diamond, just as an athlete voluntarily primes himself to begin running at the sound of the starting pistol. After that, the stimulus (either a subliminally presented shape or conscious stimulus) might in principle only have to activate that perceptual representation to trigger task-set activation. In such cases, though a subliminal stimulus might act as an ‘action trigger’ [17] releasing a voluntary, endogenously established response, this would constitute external control over actions only in a very limited sense.

Our recent study [18] circumvented this limitation by demonstrating unconscious priming of cognitive control without the need for participants to have a conscious, explicit attentional set to search for the same shapes used as primes. In Experiments 2A and 2B of that study, we introduced a learning phase to establish an association between two unconsciously-perceived instruction shapes (a diamond and a square) and two types of word task (semantic and phonological). In the learning phase, one of the two shapes was presented unconsciously (briefly presented and masked) prior to the conscious auditory instruction to perform one task or the other. Although the unconscious shapes were not instructions, the presentation of a particular unconscious shape in a trial predicted 100% of the time the task that participants would subsequently have to perform. This predictive relationship was intended to establish an association between each unconsciously presented shape and a corresponding task. Consequently, when those shapes were presented in a second, test phase, they were expected to activate the task sets with which they had been associated in the learning phase.

The shapes employed as unconscious primes were never presented as conscious task instructions or targets of any kind during either the learning or test phase. Hence, the participant should not have held an attentional set to search for those shapes. Nonetheless, when, in the test phase, an unconscious shape was presented prior to the task it had been associated with in the learning phase, it speeded performance of that task relative to when the other shape was presented, suggesting (as in Lau and Passingham's study) that the shapes had activated task sets without participants voluntarily choosing to do so in response to those shapes. These findings suggest that task-choice can be determined by unconscious external stimuli, a finding that seems to conflict with folk psychological intuition that decisions reflect ‘free’, conscious control. This conflict prompted us to consider whether the ability of such task-irrelevant stimulus associations might preferentially arise to a still greater degree if the learning and test phases were to employ consciously-perceived prime stimuli rather than brief stimuli that were not consciously discriminable. However, when we repeated the procedure presenting primes (during both test and learning phases) for 54 ms rather than 9 ms, we found no hint of an effect (16 participants; main effect of prime *F*_(1,15)_<1, n.s.); Indeed, when conscious and unconscious prime stimuli were intermixed within the same blocks of trials, we found no priming effects either of the conscious or unconscious primes.

On the basis of these pilot study findings we speculated that even minimal pairing of a prime shape with a task might exert a suppressive effect on subsequent priming of task selection by unconscious stimuli, even though the same shape is associated with a given task in the conscious and unconscious prime trials. An intuitive response to this prediction, and one that initially caused us to reject it, was that previous work such as that of Lau and Passingham (2007) and of Reuss et al. (2011) already included trials in which unconscious ‘prime’ shapes presented on some trials were also presented consciously on other trials. This might lead one to conclude that those studies had already conducted the necessary experiments to address the issues we address here. However, note that in both studies the participant held a voluntary, conscious task set to perform a particular task when presented with the very shapes (or similar shapes in Lau & Passingham's study) that were used as unconscious stimuli. That is, they had voluntary and consciously designated those shapes as task relevant (and did so throughout the study) as explicit motivations to perform one task or another. Under those conditions, it clearly would not make sense to suppress unconscious influences of external stimuli that were voluntary used as signals to control task selection.

A second objection to this prediction is that previous studies have already highlighted a disruptive role of explicit learning strategies on the learning of complex unconscious stimuli [19]. However, two features of our studies ensure that we are not studying those same phenomena. First, as we explicitly address later, manipulation of explicit awareness in our studies appears to have very different effects to those of conscious stimulus perception. Second, we only introduce conscious trials after the learning phase has taken place, so any influence of our conscious-stimulus perception manipulation must influence processes after initial learning of the prime-task relationships we assess. Our proposal is that it is simply perceiving a stimulus consciously before a task that suppresses unconscious influences on task selection: there is no requirement that participants must be explicitly aware of the predictive stimulus-task relationship.

Third, an immediate response when considering this hypothesis might be to suggest that as many types of unconscious priming can influence decisions our proposal is essentially irrelevant to the ‘freedom’ of choices. Examples of such effects are unconscious mere exposure effects [7] that influences attractiveness of stimuli with knock-on effect on choice and response priming that biases a particular motor output over others [10]–[12]. However, choice of task in the examples studied here is one step removed from such effects and need not be influenced by them. As in many previous task-switching studies, the same stimuli and responses are shared by the two tasks in a particular experiment. Under such circumstances, any biases toward particular stimuli or particular responses cannot yield a bias toward one task or another. Accordingly, there may be no net influences on task choice even when concurrent biases toward particular stimuli or responses are operating.

One final point to note is that we describe stimuli throughout this article as ‘unconscious’. These terms merely denote that the stimuli fail to satisfy our criterion for conscious discrimination (they cannot be discriminated from each other above chance even across a group of participants when later tested under single-task conditions); they nonetheless must have been perceptually processed in order to exert their measured influences on performance in our tasks. These terms of course are a useful short-hand to avoid having to repeat the phrase ‘stimuli that on subsequent test fail to meet objective criteria for awareness’ and do not imply that the stimuli themselves are (un)conscious.

Our two specific predictions regarding the effects of conscious stimulus perception on unconscious external control of high-level choices were that:

- (i)Unconscious stimuli will elicit measurable biases in performance that are consistent with those stimuli influencing task-selection processes.
- (ii)Conscious perception of the stimulus followed by selection of a task should prevent control of that choice behaviour by subsequently presented unconscious stimuli that would otherwise demonstrably exert effects.

Prediction 1 follows from previous work discussed above. Prediction 2 is novel and carries the counterintuitive connotation that once a task-irrelevant stimulus is consciously perceived prior to a task, the brain might purposefully decline to exploit the powerful predictive validity of those same stimuli when the stimuli are subsequently presented unconsciously. This prediction would not reflect an inability of the brain to exploit unconscious predictive stimuli as such ability is evident when the stimuli weren't consciously perceived. Rather, we supposed that the brain might sacrifice this benefit in order to maximise the control of task selection by conscious, endogenous processes.

The aim of the present study was therefore to measure unconscious influences in cued and free task selection, assessing whether conscious perception of prime stimuli might prevent unconscious control of task selection by task-irrelevant stimuli. Accordingly, the present study consisted of two sets of experiments representing the types of task selection: selection that is instructed by environmental stimuli and arbitrary, uncued choices. In Experiments 1 and 2, we examined instructed task selection. Experiment 1 first sought to establish that clear influences of unconscious stimuli on such behaviours were measurable under similar conditions to those employed by Zhou and Davis (2012). Finding such effects would afford an opportunity in Experiments 2A and 2B to examine unconscious influences following minimal conscious pairing of stimuli and task versus with no conscious pairing.

## Experiment 1

With one minor alteration pertaining to the use of auditory instructions, Experiment 1 replicated exactly the conditions of Zhou and Davis (2012, Experiment 2A) described above. A learning phase established associations between each of two unconsciously presented prime shapes and one of two tasks. The effects of these associations were then assessed in a test phase, when the shapes were presented, again unconsciously, prior to participants being asked (by an auditory signal) to perform one of those two tasks. We expected that when performance of a given task was preceded by unconscious presentation of a shape that had been presented prior to that task during the learning phase, the selection of, and preparation to perform, that task would be speeded, reducing average RTs to perform that task relative to when another shape had been presented.

### Methods

#### Participants

20 paid participants (6 of them male, 14 female; 18–37 years of age) were recruited in Experiment 1. The participants were healthy subjects with normal or corrected-to-normal vision, who all gave informed written consent. Ethics approval for the study was obtained from the Psychology Research Ethics Committee at the University of Cambridge.

#### Stimuli, Apparatus and Procedure

**Figure 1:**
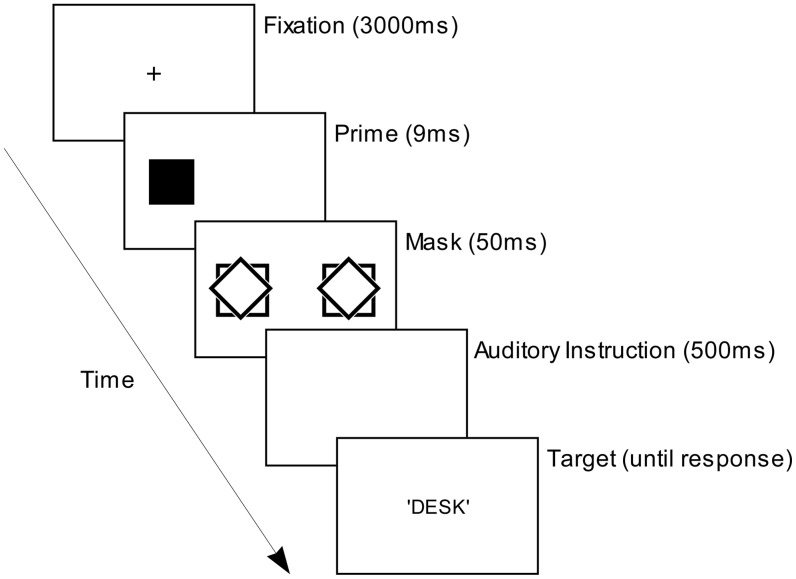
A typical trial's display sequence in learning and test phases of Experiment 1. Not illustrated are short blank (white) displays prior to the prime-, mask-, instruction- and target word displays (50, 20, 100 and 100 ms respectively).

Figure 1 overleaf schematizes a typical display sequence in a trial from the learning and test phases of Experiment 1. Following a fixation cross, a prime shape (a diamond or a square; 9 ms; subtending 1.9^°^ retinal angle vertically and horizontally) was displayed 9^°^ to the left or right of the fixation just before the presentation of two compound masks (50 ms, subtending 2^°^ retinal angle vertically and horizontally). The prime shape appeared with equal frequency and unpredictably either to the left or right of fixation. The compound masks were displayed 9^°^ to the left and right of fixation, each with edges overlapped with each prime's edges. A conscious instruction was then presented signaling which of the two tasks the participant should perform on the subsequent target word, which appeared at fixation. In the semantic task, participants judged whether the target word referred to concrete object or not and, in the phonological task, whether the word had two syllables or not (i.e. bisyllabic), pressing one of two response keys as quickly as possible to indicate their decision. To ensure minimal perceptual interference between the unconscious prime shapes and subsequent conscious instructions we used auditory signals as conscious instructions (high- 1047 Hz and low- 130 Hz pitched tones).

In the learning phase the particular unconscious prime shape presented on any trial predicted 100% of the time the task participants would subsequently be instructed to perform. This was intended to establish an association between each of the two unconscious prime shapes and one of the two tasks (either being instructed to perform the task, performing it, or both). The effects of these associations could be assessed in the test phase, in which the prime shape presented on a trial now was equally likely to occur before either task. Our prediction from our own previous findings [18] was that the presentation of unconscious prime shapes would influence the speed of preparation to perform a task and hence speed task-performance when it appeared prior to performing the same task with which it had been associated in the learning phase (these were termed ‘Congruent’ trials), *relative to* when it appeared prior to performing the other task (‘Incongruent’ trials).

On a third of trials in the test phase, neither of the unconscious prime shapes was presented. Instead, a circle, not preferentially associated with either of the word tasks in the learning phase, was presented (unconsciously). These trials were not, of course, intended to constitute an effective neutral baseline against which to compare the effects of the other prime shapes – their novelty within our procedure would necessarily have prevented them from being suitable for that purpose. Rather, as the results of Experiments 1 and 2 heavily relied on reaction times (RTs), the purpose of the neutral shapes was simply to assess whether our random selection of participants had selected participant groups with similar reaction times when neither of the task-associated prime shapes was presented; in none of the experiments were report here were there significant group differences in those trials (all F's<1, n.s.) and they were not further analyzed. Thus, on a third of trials in Experiment 1′s test phase the prime was congruent with (had been associated in the learning phase with the *same* task as) the instruction, on a third it was incongruent (had had been associated with the other task), and in the rest it was neutral (had not been associated with either task).

Combinations of prime shape, instruction tone, and task were fully counterbalanced across participants, precluding systematic associations between prime and instruction shapes. The learning phase of Experiment 1 consisted of 3 blocks of 64 trials (following 32 practice trials in which no prime was presented) and the test phase consisted of 4 blocks of 48 trials. Conscious awareness of the prime's identity was then assessed using a forced-choice discrimination task (2 blocks of 60 trials) following the test phase. In this test, the same stimulus sequences were presented as for the main task except that following the compound masks, instead of presenting a task instruction shape to signal one of the two word tasks, an instruction, ’Which shape did you see?’ was presented to signal the discrimination task. Participants attempted to identify the masked prime's shape on each trial, by pressing one of three keys accordingly.

The target words used in the experiment were selected from the Medical Research Council Psycholinguistic Database [20]. Words used in the semantic group were selected with the criteria >550 (concrete) and <350 (abstract) on the concreteness scale. In the phonological group, words containing two syllables according to the database were considered as bisyllabic, and words with one or three syllables were selected from the database as non-bisyllabic. The words were systematically counterbalanced so that words presented on half of the trials in each condition required the same motor response in both the phonological and the semantic tasks.

### Results and Discussion

Participants' accuracy in discriminating the primes' identities did not differ from chance in Experiment 1, as estimated for bias-free participants using *d′* [21], [22]. For *m*-AFC designs, discriminability is calculated as a function of accuracy and *m* [23], [24]. The average sensitivity was *d*-prime = 0.02 (SD 0.16), which did not differ significantly from zero (*t*_(19)_ = 0.44, *p* = 0.66). In our reaction time analysis of the test phase, only correct responses were assessed.

**Figure 2:**
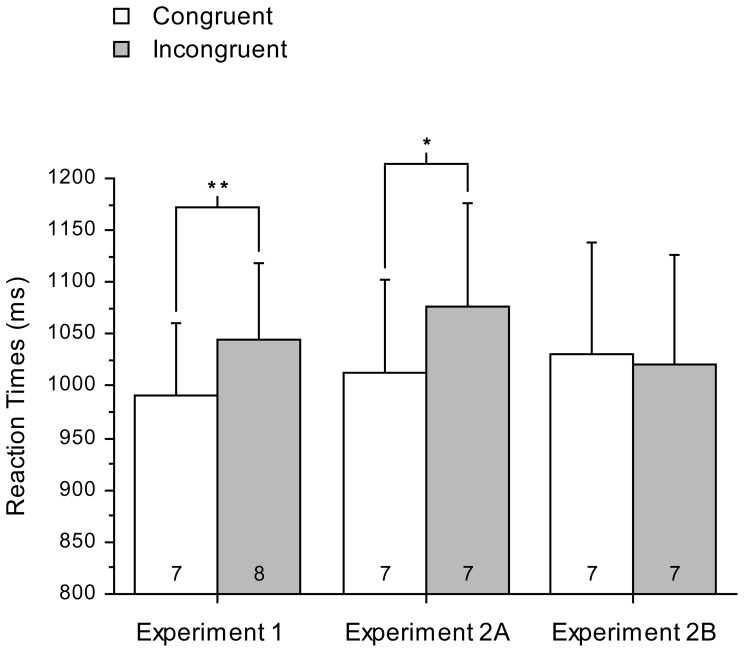
RT's for Experiments 1, 2A, and 2B. RT's (% errors; error bars 1 SEM) were plotted for trials following primes that were Congruent or Incongruent with the conscious instruction for Experiment 1, 2A and 2B.

Figure 2 (left pair of bars) plots RTs for conditions from Experiment 1 (% error rates in parentheses); note that ‘Congruent’ trials (i.e., in which prime and instruction signaled the same task) yielded faster responses than ‘Incongruent trials’ (in which prime and instruction signaled the same task) as predicted. A repeated-measures ANOVA with the factor of Prime-Instruction Congruence (Hereon ‘Congruence’; Congruent, Incongruent) yielded a main effect of Congruence, (*F*_(1,19)_ = 11.5, *p* = 0.003, partial eta squared = 0.38); RTs in congruent trials were significantly shorter than those in incongruent trials. Identical analysis of error rates yielded no significant effect that might threaten the analysis of the RTs (all *F*_(1,19)_ = <1, n.s.).

These results showed that unconscious stimuli that were never consciously presented to participants and never consciously associated with tasks in terms of instructions, could prime cued choices. As discussed earlier, such finding afforded us the opportunity to further assess the unconscious effects of prime shapes following minimal conscious presentations of them. Accordingly, in our next experiment we examined whether two conscious presentations of each prime stimulus followed by (paired with) selection and performance of a task, would preclude subsequent activation of task sets by unconscious prime stimuli.

## Experiments 2A and 2B

Experiments 2A and 2B were conducted on separate groups of participants to examine unconscious influences following minimal conscious perception in cued choices. The learning and test phases of Experiment 2A and 2B were identical to those of Experiment 1 except that four additional trials were added at the beginning of the test phase, during which each of the prime shapes was consciously presented (each shape presented consciously on two trials). In Experiment 2A, the target word that participants would usually respond to was replaced, in the four additional trials prior to unconscious test trials, with ‘XXXXX’. Participants were told to skip the trial when they saw ‘XXXXX’ by pressing either of the response keys and not to engage in either of the word tasks. In Experiment 2B, a target word was presented in the four additional trials in exactly the same manner as in unconscious test trials and participants were required to perform the word tasks.

### Methods

#### Participants

20 paid participants (7 male, 13 female; 19–34 years of age) were recruited for Experiment 2A and 20 (6 male, 14 female; 19–31 years of age) for Experiment 2B. The participants were healthy subjects with normal or corrected vision, who all gave informed written consent. Ethics approval for the study was obtained from the Psychology Research Ethics Committee at the University of Cambridge.

#### Stimuli, Apparatus and Procedure

**Figure 3:**
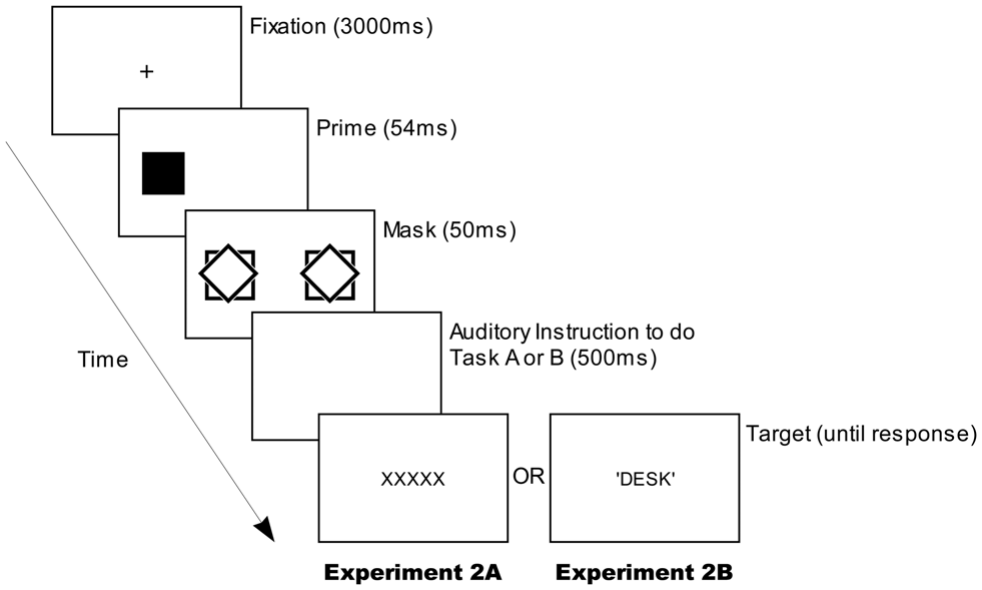
A typical trial's display sequence in four conscious trials in test phases of Experiments 2A and 2B. Not illustrated are short blank (white) displays prior to the prime-, mask-, instruction- and target displays (50, 20, 100 and 100 ms respectively).

The methods for Experiments 2A and 2B were as for Experiments 1 except for the following alterations to the test phase of each. Four weakly masked (conscious-prime) trials (as shown in Figure 3) were introduced immediately prior to the unconscious trials in test phase, as part of a single test session following a short break after the learning phase. The stimuli in conscious trials were as in unconscious trials except that prime shapes (a diamond or a square) were presented for 54 ms instead of 9 ms and the blank and masks were presented for 20 ms and 50 ms respectively. Note that only the two previously learnt prime shapes were presented in the conscious trials and not the circle ‘group comparison’ unlearnt shape, and the prime shapes presented in the conscious trials were all congruent: that is, they were always followed by tasks with which they had been previously paired in the learning phase. Each of the two previously learnt shapes was presented in two of the four conscious trials with once on each side of the screen.

In Experiment 2A, the target word was replaced with ‘XXXXX’, which was presented in the same manner and at the same spatial location as the target word. Participants were instructed prior to the start of the test phase to skip the trial if they saw ‘XXXXX’ instead of a word in by pressing either of the two response keys they used to respond to a word task in unconscious trials. In Experiment 2B, a target word was presented as in unconscious trials and participants were required to perform the word tasks in the same manner.

### Results and Discussion

Participants' average sensitivities at discriminating the primes' identities were 0.03 (SD 0.14) in Experiment 2A and 0.02 (SD 0.16) in Experiment 2B, which did not differ significantly from zero (2A- *t*_(19)_ = 0.88, *p* = 0.39; 2B- *t*_(19)_ = 0.66, *p* = 0.52). Only correct responses on unconscious trials in test phase were assessed. Identical analyses of error rates to those reported below yielded no significant main effects or interactions that might threaten the analysis of the RTs (2A- all *F*′s<1, n.s.; 2B- all *F*′s<1, n.s.). Figure 2 plots RTs for conditions from Experiment 2A and 2B (centre and right pairs of bars, respectively). Visual inspection of the plot suggests that the same effects arose in Experiment 2A as previously found in Experiment 1, and this impression was confirmed in analysis for Experiment 2A, yielding a main effect of Congruence, (*F*_(1,19)_ = 7.65, *p* = 0.012, partial eta squared = 0.29). RTs in congruent trials were significantly shorter than in incongruent trials (*t*_(19)_ = 2.77 *p* = 0.012). An identical analysis as for Experiment 1 and Experiment 2A yielded no main effect of Congruence in Experiment 2B (*F*_(1,19)_<1, n.s.). A between-subject analysis also confirmed that there was a significant difference in priming effects between Experiment 2A and 2B (*F*_(1,38)_ = 5.51, *p* = 0.024, partial eta squared = 0.13).

The results showed that, when unconscious prime shapes that had been associated with tasks were consciously presented but not paired with performance of a task, as in Experiment 2A, the unconscious influence of such stimuli in the test phase was intact (and was similar in size to that in Experiment 1). However, when such conscious presentation of prime stimuli was followed by choice behaviors (in this case, cued choices), as in Experiment 2B, the priming effects of those unconsciously learnt stimuli was diminished, though the association between prime stimuli and task was never explicitly presented. Although participants were not randomly allocated between Experiment 1 and Experiment 2 (this was only the case between Experiments 2A and 2B), an informal comparison between Experiment 1 and 2A showed that there was no significant difference in the priming effects evident in those two studies (one-way, between-s ANOVA with prime-effect in ms as the dependent variable; *F*_(1,38)_<1, n.s.). In contrast, an identical comparison between Experiment 1 and 2B resulted in a significant difference between them (*F*_(1,38)_ = 5.64, *p* = 0.023, partial eta squared = 0.13). The results further supported our prediction that when conscious presentations of primes shapes were paired with (followed by) performance of a task, they would prevent unconscious activation of task selection by those unconscious prime shapes in the test phase.

Note that in the two conscious pairings of each prime shape with a task in Experiment 2B, the prime shape always appeared prior to the task with which it was associated in the training block. Therefore those trials were not extinction trials, but rather might have been expected to strengthen further the prime-task associations established in the learning phase. Following consultation with learning theorists in our department, we believe this effect of conscious presentation is not predicted by extant models of learning and we believe it does not correspond to previously described learning phenomena. However, some other phenomena are sufficiently similar that we conducted supplementary experiments (variations of Experiment 2B) to confirm our strong suspicion that our results did not reflect those previously described processes. The three processes we assessed were: (i) long-lived inhibitory processes triggered by ignoring (the conscious prime) stimuli, (ii) effects of conscious, explicit awareness of prime-task associations that might have arisen during the four conscious trials, (iii) the labile nature of memories ‘reactivated’ by conscious presentation of a conditioned-stimulus.

Perhaps the most obvious phenomena that relate to our findings are the broad selection of poorly understood processes typified by ‘negative priming’ [25], [26]. When a stimulus is ignored as task-irrelevant, inhibition of those representations, either object representations or episodic memory representations, may remain long after the stimulus has been presented. To assess whether similar processes were responsible for the effects of conscious stimulus-task pairing observed in Experiment 2B, we re-ran that study, but altered the four trials in which the prime shapes were consciously presented. In those trials, there were now no auditory task instructions. Rather for those four trials only, the participant was instructed to perform one task if the prime shape was a square, and the other task if the prime shape was a diamond. Note that by asking participants to use the conscious prime shapes as conscious instructions, we ensured that they attended to them and treated them as task-relevant. Accordingly, no effects of ignoring those shapes during the conscious trials should arise under these new conditions.

The participants employed in this further experiment (Experiment 3A) were randomly assigned to either that study or to a second experiment (Experiment 3B). This latter study replicated the conditions of Experiment 2B, again, but ensured participants had explicit knowledge of the relationship between the prime shape presented in each trial and the task that the participant would be asked to perform. Participants were now instructed that throughout the test phase which of two shapes (presented consciously in four trials and unconsciously in the remaining trials) would be presented prior to the instruction to perform each of the two tasks (e.g., a square prior to the phonological task, a diamond prior to the semantic task). If participants' explicit learning of that relationship gleaned from the four conscious trials had resulted in the absence of a prime-shape effect in Experiment 2B, it should again have resulted in no prime effect in this new task, now that we ensured participants has that explicit knowledge. Conversely, we expected that such explicit knowledge did not result in the absence of a measured prime effect but rather would tend to strengthen the effect of the primes, being a further consciously instructed association between each prime and task.

Experiments 3A and 3B were conducted together to compare the relative effects of these two manipulations. In Experiment 3A, participants attended to the consciously presented prime shapes within the four conscious-prime trials rather than not being instructed to attend them as in Experiment 2B. In Experiment 3B, participants were aware that throughout the test phase, two particular prime shapes would be presented and that the diamond would tend to predict e.g. the phonological task and the square, the semantic task for half the participants, and the reverse pattern for the remaining participants. The relative effects of these two manipulations would then be compared to reveal whether the ‘attention’ manipulation in Experiment 3A or the ‘explicit awareness’ manipulation in Experiment 3B yielded the larger unconscious prime effects relative to the zero baseline of no detectable effect found in Experiment 2B. Second, the effects of the unconscious prime shapes in each of these new studies could then be compared statistically (though informally, in that the there was not random allocation between the participants of these new studies and the participants of Experiment 2B). For the reasons outlined above, we predicted that Experiment 3A, like Experiment 2B should yield no detectable priming effect, but that Experiment 3B should yield a priming effect.

## Experiments 3A and 3B

### Methods

#### Participants

12 paid participants (5 of them male, 7 female; 20–32 years of age) were recruited for Experiment 3A, and 12 (4 of them male, 8 female; 18–30 years of age) for Experiment 3B. The participants were healthy subjects with normal or corrected vision, who all gave informed written consent. Ethics approval for the study was obtained from the Psychology Research Ethics Committee at the University of Cambridge.

#### Stimuli, Apparatus and Procedure

The methods for Experiments 3A and 3B were as for Experiment 2B except for the following alterations to the test phase of each. In Experiment 3A, no auditory instruction was presented in the four conscious trials prior to the test phase. Participants were therefore instructed to use consciously presented prime shapes in these four trials as instruction cues to perform one of the word tasks. Auditory instructions were presented in the rest of the test trials as in Experiment 2B. In Experiment 3B, prior to the start of the test phase, participants were explicitly informed that there were two shapes briefly presented before the masks, and that these shapes would also predict the type of word tasks that would follow. Thus participants could use these shapes to help them to do the task. The test phase of Experiment 3B was identical to that in Experiment 2B.

### Results and Discussion

Participants' accuracy in discriminating the primes' identities was, in Experiment 3A, raised slightly relative to values in our previous work (averaging around 37% accuracy - chance at 33%); for the first time these values were significantly but modestly above chance (*t*_(11)_ = 4.51, *p* = 0.001). However, we concluded that this minor amount of extra awareness, although important to note, could not threaten our conclusions here. The only issue that this finding might raise for our interpretation of our findings is that the extra conscious perception in the unconscious prime trials had given rise to a suppressed effect of the primes in that study and so had affected our results. However, as our claim here is that small amounts of conscious perception (specifically in the conscious prime trials, but also generally) tend to suppress the effect of primes, such a claim would seem to echo rather than threaten our conclusions. Moreover, any minor increase in awareness showed no hint of a correlation across participants with variations in the effects of the unconscious prime shapes, so the two are unlikely related (*r*_(10)_ = 0.05, *p* = 0.87). The same assessment for Experiment 3B did not differ significantly from zero (*t*_(11)_ = 0.78, *p* = 0.45). Only correct responses on unconscious trials in test phase were assessed. Identical analyses of error rates to those reported below yielded no significant main effects or interactions that might threaten the analysis of the RTs (3A- all *F*′s<1, n.s.; 3B- all *F*′s<1, n.s.).

**Figure 4:**
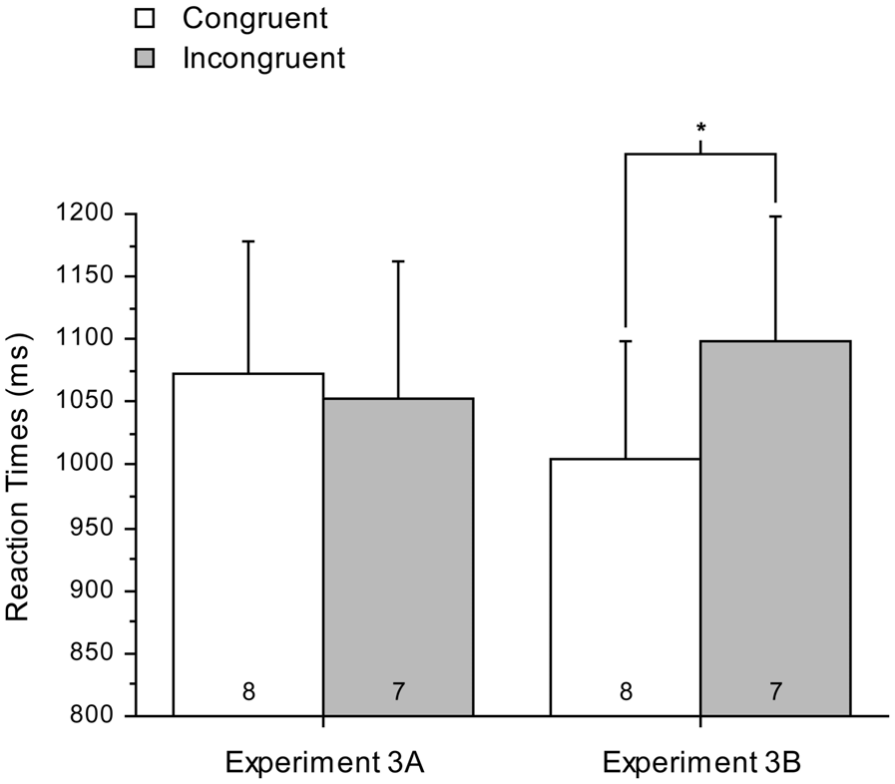
RT's for Experiment 3A and 3B. RT's (% errors; error bars 1 SEM) were plotted trials following primes that were Congruent or Incongruent with the conscious instruction for Experiment 3A and 3B.

Figure 4 plots RTs for conditions from Experiment 3A and 3B. Visual inspection of the plot suggested that the priming effects had been diminished in Experiment 3A as previously found in Experiment 2B, and this impression was confirmed in analysis for Experiment 3A, yielding no significant effect of Congruence (*F*_(1,11)_<1, n.s.). On the other hand, an identical analysis as for Experiment 2B and Experiment 3A yielded a main effect of Congruence in Experiment 3B (*F*_(1,11)_ = 6.73, *p* = 0.025, partial eta squared = 0.38). These findings indicated that the effects of conscious pairing of stimulus and task in Experiment 2B could not be ascribed either to explicit awareness of the prime shape–task associations or to ignoring of the consciously presented prime stimuli. However, one further intuitive candidate explanation of our findings remained. Perhaps the effect of our conscious prime presentations in Experiment 2B (suppressing effects of subsequently presented might have arisen because the conscious presentations acted as a recall stimulus and hence rendered learning particularly susceptible to interference. The labile nature of such learning lasts for up to 6 hours following a single presentation of a stimulus that has predicted an outcome in previous learning [27]–[29] and so would easily encompass our test phase. We considered that this explanation for our results was unlikely because typically conscious presentation of the prime stimulus without the task (as in our Experiment 2A) suffices to put learned associations into a labile state; such presentation did not cause suppression of the prime effects in that experiment, but rather only in Experiment 2B. Nonetheless, to ensure that this was not the case, we replicated Experiment 2B in a further study, Experiment 4A, which incorporated the two following amendments (to the design of Experiment 2B). First, the four brief conscious presentations were now presented after the learning phase but 6 hours prior to the test phase (this phase termed the ‘primary test phase’). Accordingly, if the four conscious presentations in Experiment 2B had merely made associations from the learning phase labile for a few hours as in previous studies, there should be no effect of those conscious presentations in this new study. Second, we now inserted an additional test phase (the ‘supplementary test phase’) after the learning phase but prior to the four conscious-prime trials. This supplementary phase was to establish that the learning phase had induced an effect of unconsciously presented primes, prior to presentation of the four conscious prime trials.

As a baseline against which to compare the effects of the unconscious prime shapes in Experiment 4′s supplementary test phase (prior to conscious prime presentations) and main test phase (6 hours after the conscious presentations), we ran a separate group of participants on a procedure that was identical but did not include any conscious presentations (Experiment 4B). Participants were randomly assigned to the two groups. 'To simplify this most complex analysis in our data, we calculated the priming effect (average RT following Incongruent prime separately for each test phase (supplementary and primary). We expected, and hoped, to find similar effects of the unconscious primes in the supplemental test phase for Experiment 4A versus for Experiment 4B. This would indicate that both groups of participants had initially had approximately the same strength of priming effect. However, we expected to find that following the presentation of four conscious-prime trials in Experiment 4A the subsequent primary test phase (6 hours later) would yield no effect of the unconscious prime shapes. In contrast, we expected that participants in Experiment 4B, who would not be presented with the prime consciously on four trials should continue to show effects of the unconsciously presented prime shapes in the primary test phase. If these expected patterns of results resulted in significantly greater unconscious prime effects in the primary test phase of Experiment 4B than Experiment 4A, this would indicate that the four conscious prime trials in Experiment 4A had significantly suppressed priming even though they arose six hours before the primary test phase. To further increase our confidence that the groups of participants in those two experiments were processing the primes similarly prior to the conscious trials we also planned to compare the priming effects in the two experiments' supplementary test phases, hoping to find no difference there.

## Experiments 4A and 4B

### Methods

#### Participants

16 paid participants (6 of them male, 10 female; 20–29 years of age) were recruited for Experiment 4A, and 16 (8 of them male, 8 female; 20–29 years of age) for Experiment 4B. The participants were healthy subjects with normal or corrected vision, who all gave informed written consent. Ethics approval for the study was obtained from the Psychology Research Ethics Committee at the University of Cambridge.

#### Stimuli, Apparatus and Procedure

The methods for Experiments 4A and 4B were as for Experiment 1 and 2B except for the following alterations. In both Experiment 4A and 4B, participants were required to participate in two test sessions with 6 hours apart. In both Experiments 4A and 4B, the first session comprised the learning and supplementary test phases (identical to those in Experiment 1). The second session, the test phase was presented again followed by an awareness test as in Experiment 1. In Experiment 4B, four conscious trials identical to those found in the test phase of Experiment 2B were presented at the end of the test phase in the first session. The second session of Experiment 4B was identical to that of Experiment 4A.

### Results and Discussion

There were two participants from each Experiment 4A and 4B, whose accuracy in discriminating the primes' identities were above chance. There was also a participant in Experiment 4B, who scored less than 70% in accuracy in the main test phase. Results from these participants were excluded from subsequent analysis. The remaining participants' accuracy in discriminating the primes' identities did not differ from chance in either experiment; *d′* average sensitivity was 0.004 (SD 0.08) in Experiment 4A and 0.04 (SD 0.13) in Experiment 4B, which did not differ significantly from zero (4A- *t*_(13)_ = 0.20, *p* = 0.84; 4B- *t*_(12)_ = 1.22, *p* = 0.25). Only correct responses on unconscious trials in test phase were assessed. Identical analyses of error rates to those reported below yielded no significant main effects or interactions that might threaten the analysis of the RTs (4A- all *F*′s<1, n.s.; 4B- all *F*′s<1, n.s.).

**Figure 5:**
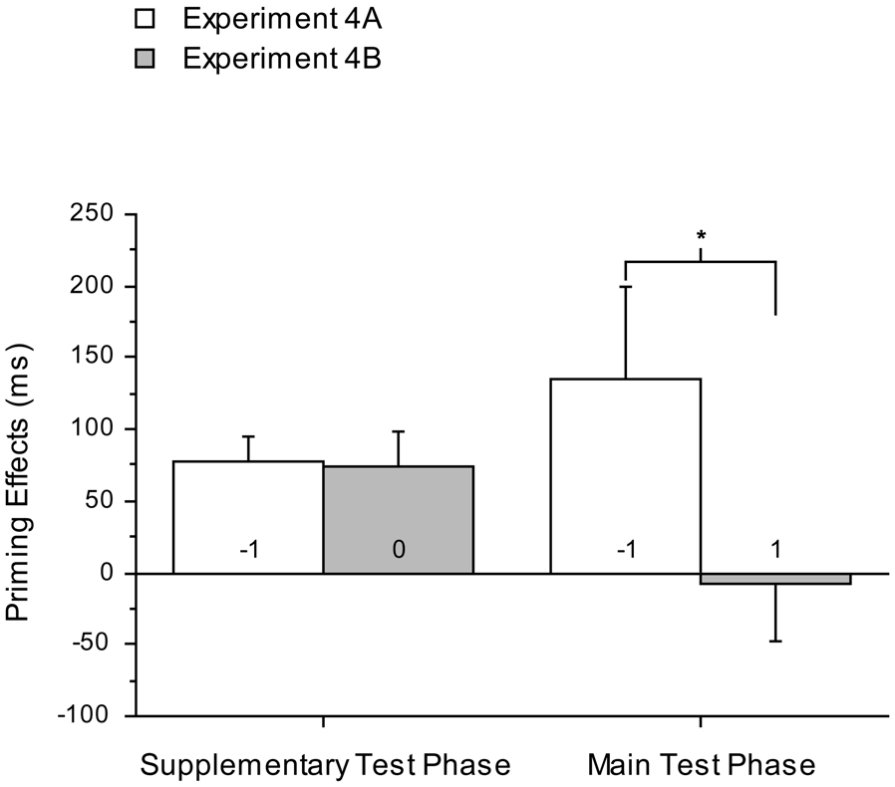
Priming effects in Experiment 4A and 4B. Priming effects (% errors; error bars 1 SEM) were plotted for both supplementary and primary test phases of Experiment 4A and 4B.

In Experiment 4A and 4B, RTs were converted into prime effects by taking differences in RTs between congruent and incongruent conditions. The priming effects in both supplementary and primary test phases for Experiment 4A and 4B were presented in Figure 5. However, the scores for the first test session of Experiment 4B were not normally distributed. Therefore, a reciprocal transformation was applied to the scores of both Experiment 4A and 4B to reduce the influence of extreme values and the transformed data were further analysed. An ANOVA analysis with factors Session and Experiment was then applied to the priming effects of Experiment 4A and 4B, which resulted in a significant interaction (*F*_(1,25)_ = 4.30, *p* = 0.049, partial eta squared = 0.15) between these two factors. Further analysis also found a significant effect of the factor Experiment in the later session (*F*_(1,25)_ = 5.12, *p* = 0.033, partial eta squared = 0.17), but not in the earlier one (*F*_(1,25)_<1, n.s.).

The results of Experiments 4A and 4B demonstrated that the suppressive effects of four conscious trial presentations are evident even when they occur six hours prior to the trials on which those effects are tested (as in the ‘primary’ test phase of Experiment 4A). This finding is inconsistent with the notion that the suppressive effects of our conscious prime presentations were due solely to those presentations rendering the associations established in the learning phase labile and subject to interference. Such a labile state would be expected to have reconsolidated within six hours, yet the suppressive effect was still evident in Experiment 4A when the conscious presentations preceded the test trials by six hours.

Having precluded three intuitive explanations of the suppressive effects of conscious prime-task pairing in Experiment 2B, we wondered whether such effects would be specific to cued task selection (triggered by conscious auditory instructions) or instead might generalise to unconscious prime effects on ‘free’ choices of task. Reuss et al. (2011) have demonstrated using a procedure based on Lau and Passingham (2007) that such free choices can be biased by unconscious prime shapes. However, to ensure that such was also the case using our procedures, we first adapted the basic procedure from Experiments 1-4 to study ‘free’ choices of task.

## Experiment 5

Experiment 5 examined the influence of unconscious stimuli in ‘free’, arbitrary choice behaviors. The learning phase of Experiment 5 was identical to that in Experiment 1. However, in the test phase, instead of presenting a conscious instruction to signal participants to perform one task or another as in Experiment 1, participants were instructed to freely choice which of the tasks they would like to perform before responding to the chosen task.

### Methods

#### Participants

16 paid participants (5 of them male, 11 female; 20–29 years of age) were recruited in Experiment 5. The participants were healthy subjects with normal or corrected vision, who all gave informed written consent. Ethics approval for the study was obtained from the Psychology Research Ethics Committee at the University of Cambridge.

#### Stimuli, Apparatus and Procedure

**Figure 6:**
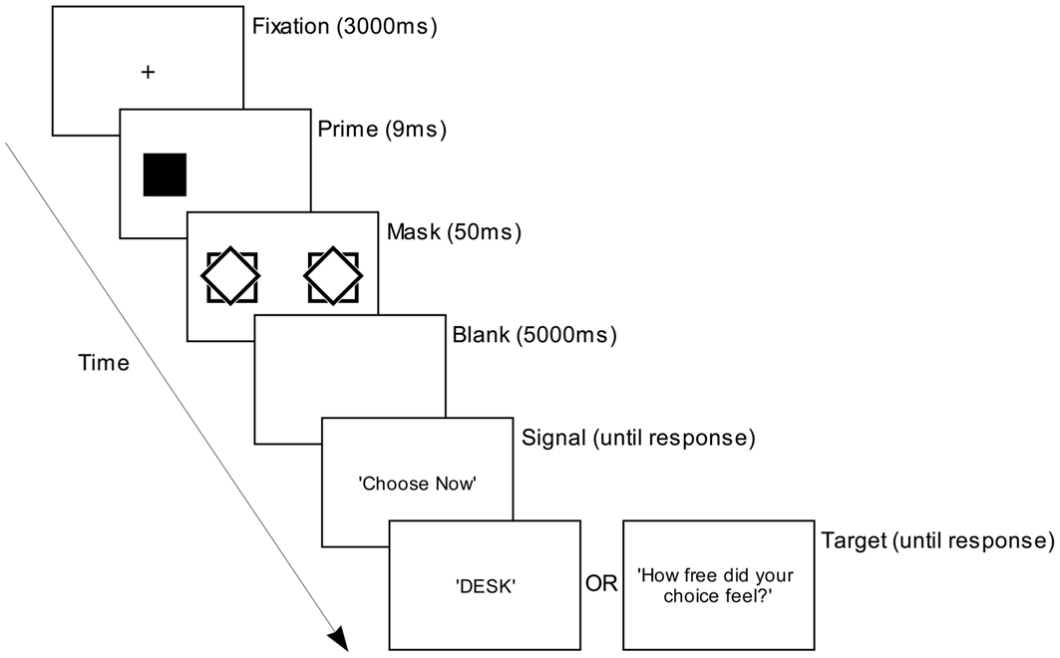
A typical trial's display sequence in test phase of Experiment 5. Not illustrated are short blank (white) displays prior to the prime-, mask- and target displays (50, 20 and 100 ms respectively).

Figure 6 schematizes a typical display sequence in a trial from the test phase of Experiment 5. The training session of Experiment 5 was identical to that in Experiment 1. The testing session of Experiment 5 was very similar to that in Experiment 1 except for the following changes. Following the masks, instead of presenting a sound instruction, a blank screen was presented for 5000 ms. Participants were instructed to choose a task they would like to perform on the subsequent target word by pressing one of two labeled keys on the keyboard, ‘S’ for a semantic task and ‘P’ for a phonological task. Participants were then presented with a signal ‘Choose Now’ on the screen to choose a task if they had not done so in 5000 ms. Once they had made the choice, a target word would be presented in the same manner as in Experiment 1, and participants needed to make judgments according to the particular task they had previously chosen; on a semantic task, participants judged whether the target word referred to concrete object or not and, in a phonological task, whether the word had two syllables or not (i.e. bisyllabic), by pressing one of two response keys as quickly as possible to indicate their decision. A word was then presented and the participants performed the task they had chosen. There was also a second type of trial, in which participants were instructed to report how free they subjectively felt their choice of task to be. In these ‘Rate Freedom’ trials, once they had pressed a key to indicate which task they would like to do, instead of presenting a target word, a question ‘How free did your choice feel?’ was presented on the screen and participants were instructed to press a key in a labeled scale on the keyboard to rate. The test phase of Experiment 5 consisted of 5 blocks of 20 trials, 80% of the trials were normal word task trials and 20% were ‘Rate Freedom’ trials. The two types of trials were randomly intermixed in the test phase. Conscious awareness of the prime's identity was assessed using a forced-choice discrimination task that was identical to the one in Experiment 1 except that only two prime shapes were presented and participants were instructed to respond by pressing one of two keys accordingly.

### Results and Discussion

Participants' average sensitivity in discriminating the primes' identities was 0.03 (SD 0.22), which did not differ significantly from zero (*t*_(15)_ = 0.46, *p* = 0.65); this corresponded to an average accuracy of 50.7%. The number of trials in which congruent task choices were made was calculated. Congruent choices arose when participants chose the task associated (in the learning phase) with a prime shape they had been presented with on a given trial. A one-sample *t*-test showed that the average proportion (54.7%) of congruent task choices made was significantly higher than chance (*t*_(15)_ = 5.83, *p*<0.001). These results indicated that an unconscious shape presented prior to a particular task during the learning phase, biased participants to choose that same task in the learning phase. That is, the effects of the primes observed in cued task-switching tasks of Experiments 1 to 4 also biased ‘free’ choices of task. We therefore next assessed whether 4 trials with conscious pairing of stimulus and task at the end of the learning phase would preclude any subsequent bias of unconscious primes on free choices of task, just as they had on cued task selection in Experiment 2B.

## Experiment 6A and 6B

The aim of Experiment 6A and 6B was to assess the role of consciousness by examining the influence of unconscious prime shapes in ‘free’, arbitrary choices following conscious presentation of the prime shapes. Experiment 6A and 6B were conducted on separate groups of participants. The learning and test phases of Experiment 6A and 6B were identical to that of Experiment 5, except that four additional trials were added at the beginning of the test phase, during which each of the prime shapes were consciously presented following the same logic and procedure in Experiment 2A and 2B. In the four additional trials prior to unconscious test trials in the test phase of Experiment 6A, ‘XXXXX’ was presented instead of a blank screen as in the later test trials and participants were instructed to skip the trial when they saw ‘XXXXX’ by pressing either of the response keys. The presentation of the X's signaled to the participant that they should not choose one task or the other on that trial. In Experiment 6B, a blank screen was presented in the four additional trials in exactly the same manner as in unconscious test trials and participants were required to perform the tasks.

### Methods

#### Participants

16 paid participants (3 of them male, 13 female; 18–32 years of age) were recruited for Experiment 6A and 16 (2 of them male, 14 female; 19–32 years of age) for 6B. The participants were healthy subjects with normal or corrected vision, who all gave informed written consent. Ethics approval for the study was obtained from the Psychology Research Ethics Committee at the University of Cambridge.

#### Stimuli, Apparatus and Procedure

**Figure 7:**
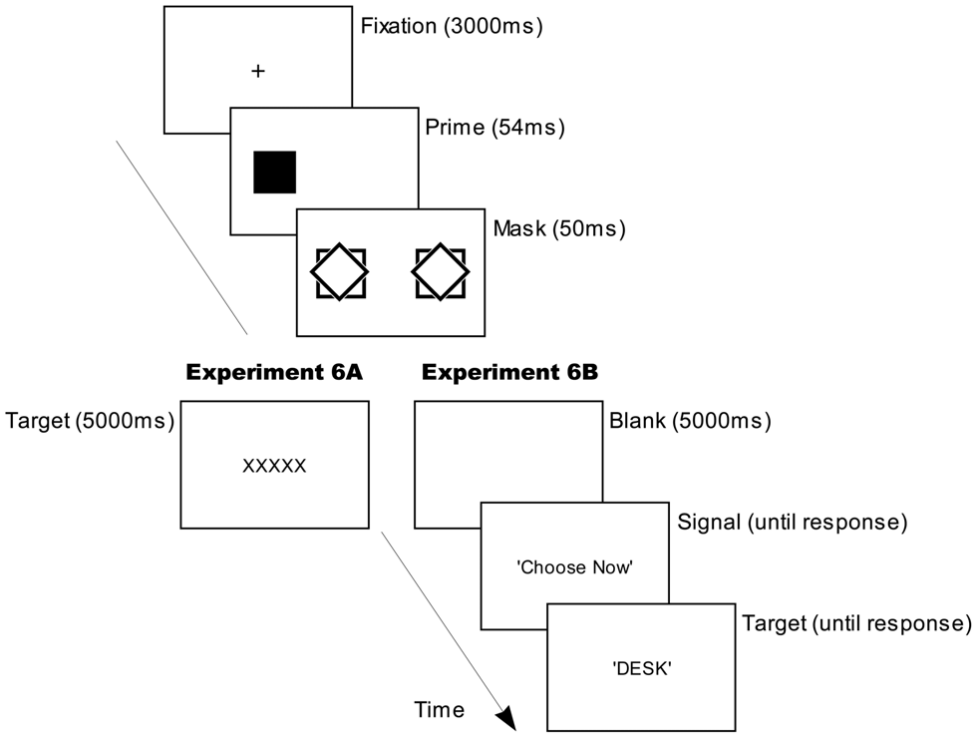
A typical trial's display sequence in four conscious trials in test phases of Experiments 6A and 6B. Not illustrated are short blank (white) displays prior to the prime-, mask- and target displays (50, 20 and 100 ms respectively).

Figure 7 schematizes a typical display sequence in a conscious trial from the test phases of Experiment 6A and 6B. The methods for Experiments 6A and 6B were as for Experiment 5 except for the following alterations to the test phase of each. Four weakly masked (conscious) prime trials were introduced prior to the unconscious prime trials in test phase. The two types of trials were run consecutively as a single test session. The stimuli in conscious prime trials were presented in the same manner as in the Experiment 2A and 2B. In Experiment 6A, the target word was replaced with ‘XXXXX’, which was presented in the same manner and at the same spatial location as the target word. Participants were instructed to skip the trial if they saw ‘XXXXX’ instead of a word by withholding responses, and the trials would run by themselves. In Experiment 6B, a target word was presented as in unconscious trials and participants were required to perform the word tasks in the same manner.

### Results and Discussion

Participants' average sensitivities at discriminating the primes' identities were 0.04 (SD 0.20) in Experiment 6A and 0.05 (SD 0.19) in Experiment 6B, which did not differ significantly from zero (6A- *t*_(15)_ = 0.90, *p* = 0.38; 6B- *t*_(15)_ = 1.13, *p* = 0.28). These sensitivities corresponded to mean accuracy of discrimination for Experiment 6A of 50.5% and for Experiment 6B of 50.8%

Choices congruent with the prime were made on 53.9% of trials, which was significantly higher than chance (*t*_(15)_ = 3.55, *p* = 0.003). However, no such prime effect was observed in Experiment 6B (*t*_(15)_<1, n.s.). An unrelated *t*-test confirmed that the prime effect was significantly smaller in Experiment 6B than Experiment 6A (*t*_(30)_ = 2.62, *p* = 0.014). The results indicated, again, that conscious presentations of prime shapes could remove unconscious influences of these prime shapes in free, arbitrary choice behaviors. However, as found in Experiment 2A and 2B, such conscious control of unconscious influences only happened when conscious presentations were immediately followed by (in this case, free) selection and performance of a task.

## General Discussion

The current findings reinforce the conclusions of previous work [18], [30], [31] that activation of task selection by unconscious stimuli can influence the efficiency of cued task selection and can bias participants to select one of two tasks in a ‘free’ choice procedure. Such influences appear to undermine the folk psychological intuition that our choices (whether cued or ‘freely’ chosen) are ultimately under our conscious, considered control rather than exogenous and unconscious processes. Indeed, these very types of influence have been cited as evidence that human adults' assumption of ‘free’ (independent of immediate environmental control) over their choices must be delusory.

A second, novel feature of our findings was that in both cued and free-choice task selection paradigms that *conscious* perception of a prime shape prior to an instruction to perform a task (and subsequent performance of that task) seems to prevent any further control of task selection by those unconscious shapes. Should this pattern of findings prove to generalise across paradigms (we have replicated it in one further set of conditions, not reported here for brevity) this would suggest that unconscious stimuli can control task selection processes only when they are either exclusively unconscious (not conscious on any trials) or explicitly treated as task relevant by the participant. Task-irrelevant, unconscious stimuli that sometimes achieve conscious perception will likely not influence task selection when presented unconsciously; conscious perception of them on some trials would prevent their influence as they did in the studies reported here (indeed, a further study, not reported here intermixed trials in this unpredictable manner and found no influence of the unconscious stimuli).

At first glance this latter conclusion might not seem to impact the widespread assumption that unconscious stimuli can control task selection. However, we suggest that it greatly narrows the ranges of stimuli in everyday viewing conditions that will be able to control choices. Only task-irrelevant stimuli that are reliably perceived unconsciously such that they can become associated with and influence task selection, yet are *never* consciously perceived (resulting in suppression of that learning) will be very rare events indeed. Accordingly, our current findings suggest that, beyond the highly controlled conditions of the laboratory, task choices may generally approach independence from unconscious environmental stimuli. That is, our laboratory findings seem broadly to accord with participants' own subjective assessment that their choices are not controlled by stimuli within the immediate environment. Accordingly, these results raise the possibility that participants' assumptions about their own autonomy from immediate environmental control may veridically (to a degree) reflect their autonomy outside the laboratory and only reflect inaccurately environmental control of their decisions within the laboratory- conditions of which they have limited experience.

A second set of limitations of the current work concerns our focus on a limited range of conditions. Counter-examples to our general conclusion likely hold, in particular when selection of a stimulus-task association has become strongly consolidated and is habitual (for example, contextual cues in cases of addiction). Note, however, that in many such cases, many such stimuli will have been treated consciously as task relevant at some stage and are therefore only examples of unconscious control in a more limited sense (see our discussion in the Introduction). Finally, our new results are limited in that they do not speak directly to the most common types of choice examined by studies of ‘free willed choices’ [2], [32], [33], namely simple choices as to which finger to flex. Although we feel that examining task rather than digit selection confers some advantages, it remains unclear whether our results will generalise to those other choices.

Despite these limitations, our findings are exciting in that they are not predicted by learning theory (our conscious trials would normally be expected either to further reinforce stimulus-task associations formed in the learning phase, or at least, not to suppress that learning more than the extinction trials constituted by our conscious-stimulus alone (with no task) control condition). Rather, they seem to be more consistent with the notion that a process related to conscious pairing of the stimulus and task (but not explicit awareness) in our experiments, operates to limit environmental control of task selection, even by unconscious stimuli. This process is rather reminiscent of previous report by [34] that perceptual learning arises for subthreshold task-irrelevant motion signals but not for supretheshold (conscious) signals. However, that finding differs from ours in two key respects. First, in our experiments, the conscious trials suppress the effects of unconscious stimuli. This cannot have happened in the Tsushima study as given their interleaved conscious and unconscious stimulus trials, an analogous process to that revealed here would have prevented learning of the unconscious stimuli too. Second, in our study, the effect of the conscious presentations was amnesic- it arose after the learning had arisen rather than (as Tsushima et al. concluded) affecting learning. Third, the effect of our brief conscious presentations eradicates all evidence of learning 6 hours later after the briefest of conscious ‘glimpses’: it seems to reflect a permanent suppression of learning rather than momentary, trial-by-trial suppression. We therefore suggest that our results may reveal a new potential functional role for processes associated with conscious perception in limiting control of task selection by task-irrelevant unconscious stimuli.
